# Resistance Training Variables for Optimization of Muscle Hypertrophy: An Umbrella Review

**DOI:** 10.3389/fspor.2022.949021

**Published:** 2022-07-04

**Authors:** Roberto Bernárdez-Vázquez, Javier Raya-González, Daniel Castillo, Marco Beato

**Affiliations:** ^1^Faculty of Health Sciences, Universidad Isabel I, Burgos, Spain; ^2^Valoración del Rendimiento Deportivo, Actividad Física y Salud, y Lesiones Deportivas (REDAFLED), Universidad de Valladolid, Soria, Spain; ^3^School of Health and Sports Science, University of Suffolk, Ipswich, United Kingdom; ^4^Institute of Health and Wellbeing, University of Suffolk, Ipswich, United Kingdom

**Keywords:** muscle mass, cross sectional area, load magnitude, training methods, resistance

## Abstract

This umbrella review aimed to analyze the different variables of resistance training and their effect on hypertrophy, and to provide practical recommendations for the prescription of resistance training programs to maximize hypertrophy responses. A systematic research was conducted through of PubMed/MEDLINE, SPORTDiscus and Web of Science following the preferred reporting items for systematic reviews and meta-analyses statement guidelines. A total of 52 meta-analyses were found, of which 14 met the inclusion criteria. These studies were published between 2009 and 2020 and comprised 178 primary studies corresponding to 4,784 participants. Following a methodological quality analysis, nine meta-analyses were categorized as high quality, presenting values of 81–88%. The remaining meta-analyses were rated as moderate quality, with values between 63–75%. Based on this umbrella review, we can state that at least 10 sets per week per muscle group is optimal, that eccentric contractions seem important, very slow repetitions (≥10 s) should be avoided, and that blood flow restriction might be beneficial for some individuals. In addition, other variables as, exercise order, time of the day and type of periodization appear not to directly influence the magnitude of muscle mass gains. These findings provide valuable information for the design and configuration of the resistance training program with the aim of optimizing muscle hypertrophy.

## Introduction

Hypertrophy is defined as an increase in muscular size, which can be achieved through exercise. Two main factors contribute to this physiological phenomenon such as sarcoplasmic hypertrophy (i.e., increased muscle glycogen storage) and myofibrillar hypertrophy (i.e., increased myofibril size and myofibrillar number) (Triplett and Haff, 2015). In this regard, resistance training is considered the gold standard for increasing muscle mass, which is based on three key variables such as mechanical stress, metabolic stress, and muscle damage (Ahtiainen et al., 2003). Traditionally, resistance training focused on hypertrophy is characterized by moderate load, high total volume load and short rest periods (Kraemer and Ratamess, 2005), although the effects of resistance programs vary depending on the manipulation of its variables (Schoenfeld and Grgic, 2017). Since promising effects related to the increase of muscular size on both performance and health have been previously reported (Maestroni et al., 2020), it seems justified to search for the most effective methods to generate hypertrophy.

Due to the strong positive relationship observed between the muscle's capacity to generate force and their cross-sectional area (CSA) (Maughan et al., 1983), hypertrophy is one of the main goals pursued by both professional and recreational athletes. Thus, several research studies have analyzed the effects of resistance training on hypertrophy and its subsequent force level (Hornsby et al., 2018). However, it is important to highlight that most team sports require high-force level, but also that the athletes must apply it in the minimum time period (Taber et al., 2016). Regarding this, increasing muscle mass include a positive influence on rate of force development and power, which improve sportive actions such sprinting, jumping, and change of direction ability (Keiner et al., 2014; Seitz et al., 2014; Suchomel et al., 2018). In addition, muscle mass is a key factor in sports disciplines where the quality and quantity of muscle development is judged, such as bodybuilding (Schoenfeld, 2010). Therefore, promoting hypertrophy could be a relevant strategy for improving sports performance (Andersen et al., 2000).

From a human health standpoint, muscle mass plays a significant role in several actions of daily life as locomotion (McLeod et al., 2016), so low levels of muscle mass may lead to an increased risk of several diseases (Maestroni et al., 2020). In this regard, resistance training and their associated hypertrophy adaptations have been shown to have health benefits such as reducing body fat, increasing metabolic rate, lowering blood pressure and cardiovascular demands on exercise, improving blood lipid profile, glucose tolerance and insulin sensitivity, a reduction in the risk of suffering from type II diabetes, an improvement in mobility and functional capacity, an increase in strength, muscle and bone mass, and an increase in related factors with quality of life (Wolfe, 2006; Maestroni et al., 2020). Specifically, Balachandran et al. (2014) applied a hypertrophy-oriented resistance program (3 sets of 10–12 repetitions using 70% of their one-repetition maximum and 1–2 min recovery) with sarcopenic obese adults during 15 weeks obtaining improvements in functional capability and power, as well as a reduction in fat mass. On the other hand, Kadoglou et al. (2012) observed significant improvements in glycemic control, insulin sensitivity and triglycerides after the application of a hypertrophy training program (2–3 sets of 8–10 repetitions using 60–80% of the one-repetition maximum and 1–2 min recovery) in adults with type 2 diabetes mellitus. For all these aforementioned benefits, a comprehensive and controlled increase in muscle mass seems to be recommended for anyone, regardless of their age or fitness level.

Muscle hypertrophy adaptations can be obtained through several resistance training programs (Lixandrão et al., 2015; Radaelli et al., 2015; Fink et al., 2016). However, there is no well-established consensus on how resistance training variables should be manipulated to optimize muscle growth, so an umbrella review on this topic is necessary. An umbrella review is characterized by a unique criterion for the selection of scientific evidence, which only considers for inclusion the higher standard of evidence such as systematic reviews and meta-analyses (Aromataris et al., 2015). This approach offers the opportunity to compare and discuss findings of different review papers—that can be summarized in a single review. Thus, the aims of this review were, firstly, to analyze the current and high-quality scientific literature (i.e., meta-analysis) on the manipulation of different variables of resistance training and their effect on hypertrophy responses, and, secondly, to provide practical recommendations for the prescription of resistance training programs to maximize hypertrophy responses.

## Methods

### Umbrella Review Design

The present umbrella review was carried out following the guidelines set forth by the working group of Aromataris et al. (2015) and followed the Preferred Reporting Items for Systematic Reviews and Meta-analysis (PRISMA) statement guidelines (Page et al., 2021).

### Search Strategy

For this research, the following database were included: PubMed/MEDLINE, SPORTDiscus and Web of Science. Likewise, ResearchGate was used as a source of complementary information. The search syntax included the following keywords coupled with Boolean operators: “meta-analysis” AND (“resistance training” OR “resistance exercise” OR “strength training” OR “strength exercise” OR “strengthening exercise” OR “weight lifting” OR “weight training” OR “blood flow restriction” OR “blood-flow restricted” OR “blood flow restricted” OR “blood restriction” OR BFR OR hypoxia OR “muscle actions”) AND ((hypertrophy OR muscles OR CSA OR “cross sectional area” OR “cross-sectional area” OR growth OR “muscle size” OR “muscle thickness” OR “lean body mass” OR LBM OR “fat free mass” OR “fat-free mass” OR “skeletal muscle” OR “muscle fibers” OR bodybuilding OR “body building” OR “muscle gain” OR “muscular volume” OR “body composition” OR “muscular adaptations” OR “hypertrophic effects”) AND (volume OR frequency OR frequencies OR sets OR multiple OR single OR tempo OR velocity OR speed OR duration OR repetitions OR order OR “split training” OR “total body training” OR “split routine” OR “split weight training” OR ((training OR light OR low OR “low-” OR “high-”) AND load) OR “low-load” OR “high-load” OR intensity OR eccentric OR concentric OR shortening OR lengthening OR “contraction mode” OR “time-of-day” OR “time of day” OR “diurnal fluctuations” OR “circadian variation” OR “circadian rhythms” OR program OR programs). A secondary search was performed based on the screening of the reference lists of the selected meta-analyses. The last and definitive search was conducted on 27th November 2021. Two authors (RBV and JR) independently screened the title and abstract of each reference to locate potentially relevant studies and reviewed them in detail to identify articles that met the inclusion criteria. Any discrepancies between the authors in the selection process were solved in consultation with a third reviewer (DC).

### Inclusion Criteria

Meta-analyses published in English whose aim was to analyze the effect of manipulating different variables of resistance training in muscle hypertrophy adaptations were included in this umbrella review. Following to the Participant-Intervention-Comparison-Outcome (PICO) process for evidence-based practice (Schardt et al., 2007), the subsequent inclusion criteria were applied:

a) *Participants:* Male and/or female healthy and physically active practitioners. Studies focused on specific age-populations as children or elderly participants were excluded.
b) *Interventions:* Resistance training programs with traditional materials (i.e., free weights and weight stack machines).
c) *Comparison group:* Usual training (no additional training).
d) *Outcome measures:* Muscle mass, CSA, lean body mass, muscle girth, muscle thickness, fat-free mass, muscle fibers and muscle volume.

### Methodological Quality Analysis

The methodological quality of the included meta-analyses was assessed through the Assessing the Methodological Quality of Systematic Reviews 2 (AMSTAR 2) checklist, which is considered as a reliable and valid tool to evaluate the risk of bias (Shea et al., 2017). AMSTAR 2 is composed by 16 different items, which were answered with a “yes”, “no”, “cannot answer” or “not applicable” and only positive answers (i.e., “yes”) allow to sum 1 point. Meta-analyses were classified as high quality (at least 80% of the items were satisfied), moderate quality (between 40 and 80% of the items were satisfied) or low quality (<40% of the items were satisfied) attending to the obtained score in the AMSTAR 2 checklist.

### Quality of the Evidence Evaluation

The quality of the evidence was evaluated using the modified Grading of Recommendations Assessment, Development and Evaluation (GRADE) principles (Guyatt et al., 2011). In this sense, systematic reviews were classified as high (i.e., at least two high-quality primary studies), moderate (i.e., at least one high quality primary study or at least two moderate-quality primary studies), low (i.e., only moderate-quality primary studies and/or inconsistent results in the primary studies) or very low (i.e., no medium to high quality systematic review identified on this topic). If the quality of the primary studies was not assessed, the systematic review must be classified as “no evidence from systematic review”.

### Study Coding and Data Extraction

The following moderator variables were extracted from the included reviews: (a) authors and year of publication, (b) resistance training variable analyzed, (c) main aim of the meta-analysis, (d) number of studies/participants included in the meta-analysis, (e) mean interventions duration, (f) heterogeneity among primary studies (I^2^), and (g) main findings or conclusions reported by the authors. Data extraction, methodological quality assessment and quality of the evidence evaluation were performed independently by two authors (RBV and JRG) and discrepancies between the authors were resolved in consultation with a third reviewer (DC).

## Results

### Search Results

**Figure 1:**
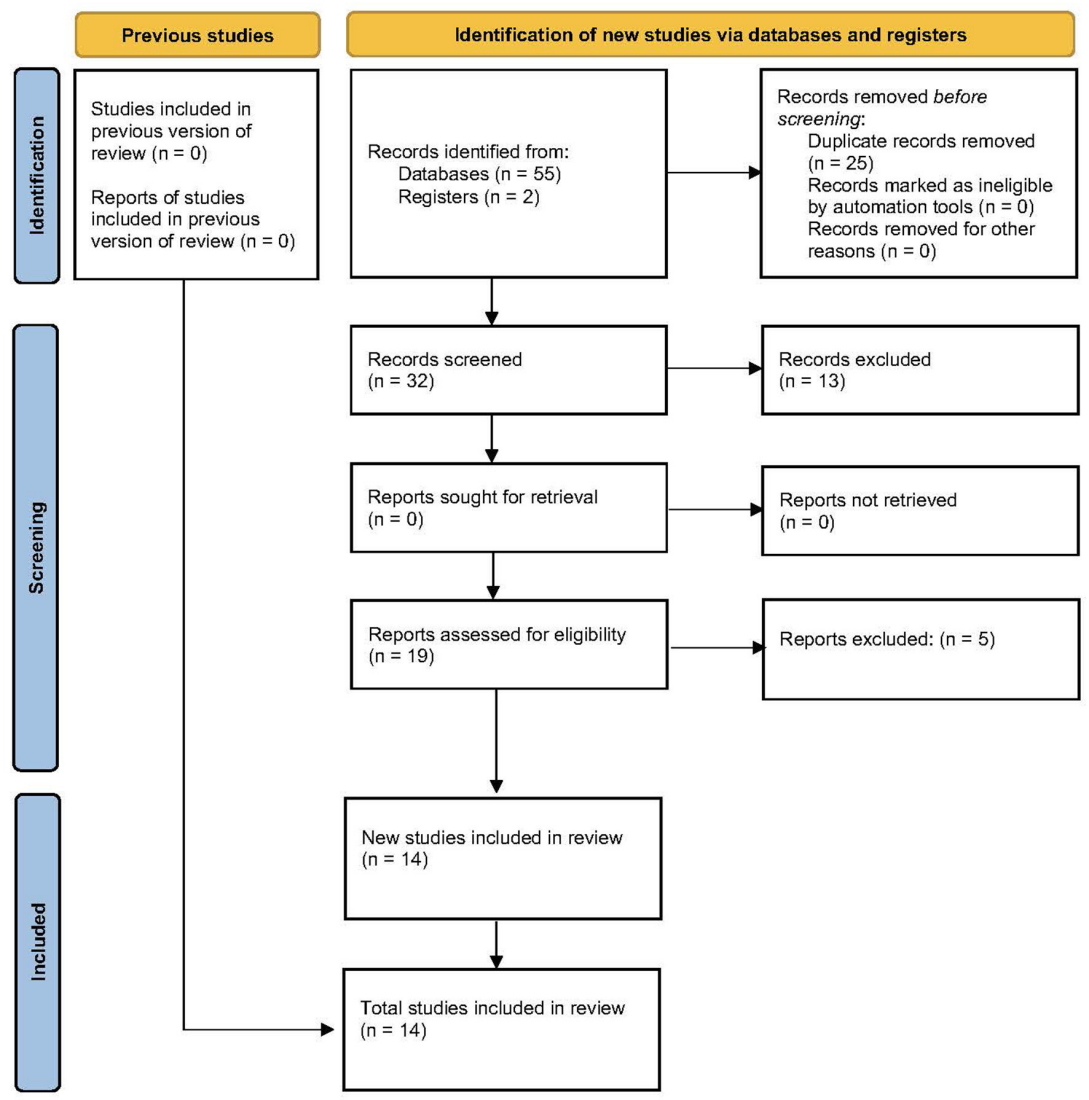
Flow diagram of the study retrieval process.

Figure 1 shows the flow diagram of the meta-analyses' retrieval process followed in this umbrella review. The initial search identified 55 meta-analyses, while 2 additional meta-analyses were found through the secondary search. Subsequently, 25 duplicate records were removed, and 13 meta-analyses were excluded based on their titles and/or abstracts. Nineteen meta-analyses were read in more detail (i.e., full-text) and 14 meta-analyses were included in the umbrella review (Roig et al., 2009; Krieger, 2010; Schoenfeld et al., 2015, 2016a, 2017a,b,c, 2019a; Slysz et al., 2016; Grgic et al., 2017, 2019; Lixandrão et al., 2018; Grgic, 2020; Nunes et al., 2020).

### Descriptive Characteristics of the Umbrella Review

The included meta-analyses are summarized in Table 1. These studies were published between 2009 and 2020 and comprised 178 primary studies corresponding to 4,704 participants. The 14 selected meta-analyses were classified attending to the analyzed variable, differentiating between volume (Krieger, 2010; Schoenfeld et al., 2017a), frequency (Schoenfeld et al., 2019a), intensity (Schoenfeld et al., 2016a, 2017c; Grgic, 2020), contraction type (Roig et al., 2009; Schoenfeld et al., 2017b), repetition duration (Schoenfeld et al., 2015), exercises order (Nunes et al., 2020), time of day (Grgic et al., 2019), periodization followed (Grgic et al., 2017) and blood-flow restriction (Slysz et al., 2016; Lixandrão et al., 2018).

**Table 1:** Summary of meta-analyses that investigated the effects of modify resistance training variables on hypertrophy.

**References**	**Variable**	**Aim**	**Studies (participants)**	**Interventions' duration**	**Heterogeneity (I^**2**^)**	**Findings/conclusions**
Krieger (2010)	Volume	To compare the effects of single and multiple sets per exercise on muscle hypertrophy.	8 (322)	From 6 to 24 weeks	NR	Multiple sets (2–3 sets) are associated with 40% greater hypertrophy-related than 1 set, in both trained and untrained subjects. the trend was observed that 4–6 sets could give an even greater response, but the small number of included studies prevents from establishing any definitive conclusions.
Schoenfeld et al. (2017a)	Volume	To elucidate the effects of total weekly volume on changes in measures of muscle mass.	15 (390)	From 6 weeks to 6 months	NR	Although low volumes ( ≤ 4 weekly sets/muscle group) are enough to get substantial gains in muscle hypertrophy, the findings indicate a graded dose-response relationship whereby increases in volume produce greater gains in muscle hypertrophy.
Schoenfeld et al. (2019a)	Frequency	To investigate the effects of weekly training frequency on hypertrophic adaptations.	25 (836)	From 6 to 30 weeks	0%	Resistance training frequency does not significantly or meaningfully impact muscle hypertrophy when volume is equated. Conversely, a significant effect favoring higher frequencies were observed when volume was not equated.
Schoenfeld et al. (2016a)	Intensity	To compare the effects of low- vs. high-load training in enhancing post-exercise muscular hypertrophy.	8 (191)	From 6 to 13 weeks	NR	Training with loads ≤ 60% 1RM can promote substantial increases in muscle hypertrophy in untrained individuals, and although a strong trend was noted for superiority of heavy loading regarding to muscle hypertrophy, no significant differences were observed.
Schoenfeld et al. (2017b)	Intensity	To compare changes in strength and hypertrophy between low- vs. high-load resistance training protocols.	10 (630)	From 6 weeks to 1 year	NR	The findings indicate that changes in measures of muscle hypertrophy were similar when trained with low-loads compared to high-loads.
Grgic (2020)	Intensity	to explore the effects of low-load vs. high-load resistance training on type I and type II muscle fiber hypertrophy.	10 (120)	From 6 to 12 weeks	0-18%	This meta-analysis did not provide significant differences in hypertrophy when compared the effects of low-loads vs. high-loads (performed to momentary muscular failure) in both type I and type II muscle fibers.
Roig et al. (2009)	Contraction type	To determine if eccentric exercise is superior to concentric exercise in stimulating gains in muscle mass.	20 (678)	From 4 to 25 weeks	65%	Eccentric training appears to be more effective at increasing muscle mass than concentric training, maybe due to the higher forces developed during this type of exercise. Additionally, adaptations after eccentric training are highly specific to the velocity.
Schoenfeld et al. (2017c)	Contraction type	To compare the hypertrophic effects of concentric vs. eccentric training in healthy adults following regimented resistance training.	15 (356)	From 6 weeks to 5 months	NR	Although both concentric and eccentric modes promote significant muscular hypertrophy, a small advantage favoring eccentric training was observed for promoting a hypertrophic response.
Schoenfeld et al. (2015)	Repetition duration	To determine whether alterations in repetition duration can amplify the hypertrophic response to resistance training.	8 (239)	From 6 to 14 weeks	NR	Similar gains in hypertrophy were observed when training with repetition durations ranging from 0.5 to 8 s to concentric muscular failure. In addition, training at volitionally very slow durations (10 s per repetition) is inferior from a hypertrophy standpoint.
Nunes et al. (2020)	Exercises order	To analyze the effects of exercise order on muscular hypertrophy.	11 (268)	From 6 to 12 weeks	0%	The findings obtained indicated that gains in muscle hypertrophy are not influenced by the exercise order (multi-single vs. single vs. multi) within resistance training programs.
Grgic et al. (2019)	Time of day	To elucidate the effects of morning vs. evening resistance training on muscle hypertrophy.	6 (221)	From 6 to 24weeks	0%	The findings showed that increases in muscle size are similar irrespective of the time of day at which the training is performed.
Grgic et al. (2017)	Periodization	To compare the effects of linear periodization and daily undulating periodization resistance training programs on muscle hypertrophy.	13 (303)	From 6 to 26 weeks	NR	The findings obtained indicated that the effects of the two periodization models (lineal and undulating) on muscle hypertrophy are likely to be similar.
Slysz et al. (2016)	BFR	To assess the effectiveness of BFR exercise on muscle hypertrophy compared to traditional resistance training.	19 (377)	NR	NR	The findings showed that suggests that the addition of BFR to dynamic exercise training is effective for augmenting changes in muscle size.
Lixandrão et al. (2018)	BFR	To compare the effects of high-load vs. low-load resistance training associated with BFR on muscle gains.	10 (222)	From 4 to 12 weeks	NR	The results obtained demonstrate similar muscle gains for high-load as compared with low-load resistance training associated with BFR.

*NR, non-reported; 1RM, one repetition maximum; BFR, blow flood restriction*.

### Methodological Quality Assessment and Quality of the Evidence Evaluation

The methodological quality of the 14 included meta-analyses is presented in Table 2. Nine meta-analyses were categorized as high quality, presenting values of 81 and 88% (i.e., 13 items satisfied) (Schoenfeld et al., 2015, 2017a,b, 2019a; Grgic et al., 2017, 2019; Lixandrão et al., 2018; Nunes et al., 2020). The remaining **meta-analyses** were rated as moderate quality, with values between 63 and 75% (i.e., from 10 to 12 items satisfied) (Roig et al., 2009; Krieger, 2010; Schoenfeld et al., 2016a, 2017c; Slysz et al., 2016; Grgic, 2020). According to GRADE, 8 meta-analyses were based on high-quality primary studies (i.e., high GRADE) (Roig et al., 2009; Slysz et al., 2016; Grgic et al., 2017, 2019; Schoenfeld et al., 2017c; Lixandrão et al., 2018; Grgic, 2020; Nunes et al., 2020) while the other 7 meta-analyses did not presented information regarding to quality (Krieger, 2010; Schoenfeld et al., 2015, 2016a, 2017a,b, 2019a).

**Table 2:** Overall results of the AMSTAR 2 checklist.

**References**	**1**	**2**	**3**	**4**	**5**	**6**	**7**	**8**	**9**	**10**	**11**	**12**	**13**	**14**	**15**	**16**	**Score**	**GRADE**
Krieger (2010)	Yes	No	Yes	Yes	No	No	No	Yes	Yes	No	Yes	Yes	Yes	Yes	Yes	Yes	69% moderate	NEFSR
Schoenfeld et al. (2017a)	Yes	No	Yes	Yes	Yes	Yes	No	Yes	Yes	No	Yes	Yes	Yes	Yes	Yes	Yes	81% high	NEFSR
Schoenfeld et al. (2019a)	Yes	No	Yes	Yes	Yes	Yes	No	Yes	Yes	No	Yes	Yes	Yes	Yes	Yes	Yes	81% high	NEFSR
Schoenfeld et al. (2016a)	Yes	No	Yes	Yes	Yes	Yes	No	Yes	Yes	No	Yes	Yes	Yes	Yes	Yes	No	75% moderate	NEFSR
Schoenfeld et al. (2017b)	Yes	No	Yes	Yes	Yes	Yes	No	Yes	Yes	No	Yes	Yes	Yes	Yes	Yes	No	75% moderate	High
Grgic (2020)	Yes	No	Yes	Yes	No	No	No	Yes	Yes	No	Yes	Yes	Yes	Yes	Yes	No	63% moderate	High
Roig et al. (2009)	Yes	No	Yes	Yes	No	No	No	Yes	Yes	No	Yes	Yes	Yes	Yes	Yes	Yes	69% moderate	High
Schoenfeld et al. (2017c)	Yes	No	Yes	Yes	Yes	Yes	No	Yes	Yes	No	Yes	Yes	Yes	Yes	Yes	Yes	81% high	NEFSR
Schoenfeld et al. (2015)	Yes	No	Yes	Yes	Yes	Yes	No	Yes	Yes	No	Yes	Yes	Yes	Yes	Yes	Yes	81% high	NEFSR
Nunes et al. (2020)	Yes	No	Yes	Yes	Yes	Yes	No	Yes	Yes	No	Yes	Yes	Yes	Yes	Yes	Yes	81% high	High
Grgic et al. (2019)	Yes	No	Yes	Yes	Yes	Yes	No	Yes	Yes	No	Yes	Yes	Yes	Yes	Yes	Yes	81% high	High
Grgic et al. (2017)	Yes	No	Yes	Yes	Yes	Yes	No	Yes	Yes	No	Yes	Yes	Yes	Yes	Yes	Yes	81% high	High
Slysz et al. (2016)	Yes	No	Yes	Yes	Yes	No	No	No	Yes	No	Yes	Yes	Yes	Yes	Yes	Yes	69% moderate	High
Lixandrão et al. (2018)	Yes	No	Yes	Yes	Yes	Yes	No	Yes	Yes	No	Yes	Yes	Yes	Yes	Yes	Yes	81% high	High

*AMSTAR 2, Assessing the Methodological Quality of Systematic Reviews 2; NEFSR, No evidence from systematic review*.

## Discussion

The main aims of this review were, firstly, to analyze the different variables of resistance training and their effect on hypertrophy responses, and secondly, to provide practical recommendations for the prescription of resistance training programs to maximize hypertrophy responses. Based on the 14 meta-analyses and the 178 primary studies included in our umbrella review, we can conclude that the variables of volume, frequency, intensity, contraction type, repetition duration, and the application of the restriction of blood flow can generate hypertrophy adaptations in healthy subjects. Conversely, other variables such as exercise order, time of the day and type of periodization appear not to directly influence the magnitude of muscle mass gains, however further research is necessary to clarify their capability to stimulate hypertrophy. The findings reported in this umbrella review provide valuable information for the design and configuration of resistance training programs aiming at optimizing muscle hypertrophy.

### Sets

Volume is commonly defined as the total amount of work performed (Schoenfeld and Grgic, 2017) and can be expressed as the total number of sets/repetition per exercise (Wernbom et al., 2007; Schoenfeld et al., 2017a) or the total number of repetitions multiplied by the amount of weight used in an exercise across sets (Schoenfeld et al., 2016b). This variable has received a great deal of attention with respect to enhancing muscle hypertrophy (Schoenfeld and Grgic, 2017), since it has been traditionally assumed that prescribing high-volume during resistance training programs will produce greater gains in muscle mass (McCall et al., 1999). This statement is supported by the fact that, when the rest of the variables remain constant, increases in volume will necessarily increase the overall time-under-tension, which has been proposed as an important driver of anabolism (Burd et al., 2012). However, it is still not clear if there is a real dose-response relationship for this variable and if there is a cut-off point from which, even if the volume increases, the muscle hypertrophy does not increase. In this regard, Krieger (2010) performed a meta-analysis in order to compare the effects on hypertrophy response between the use of one and multiple sets per exercise and to establish a dose-response relationship between training volume and hypertrophy adaptations. This author found that performing multiple sets (2–3 sets) entails 40% more hypertrophy compared to a single set (Krieger, 2010). Regarding the analysis of the dose-response relationship, the results of this meta-analysis (Krieger, 2010) reported significant differences in muscle mass gains when 2-3 sets compared to 1 set were performed, while no differences were observed when comparing volumes of 2–3 sets vs. 4–6 sets. This could be due to the theoretically increasing of protein synthesis with increased volume up (Spangenburg, 2009) to a point (Kumar et al., 2009), from which this production remains stable—limiting greater gains in muscle mass. According to the previous study, Schoenfeld et al. (2017a) observed a graded dose-response relationship between resistance training weekly volume and muscle growth. Specifically, these authors established that low-volume protocols ( ≤ 4 weekly sets per muscle group) could be enough to get substantial gains in muscle hypertrophy, which is valuable information to those for which the conservation of energy is an ongoing concern or those with a reduced time availability. However, they also observed that at least 10 weekly sets per muscle group is necessary to maximize increases in muscle mass (Schoenfeld et al., 2017a). Some authors hypothesized that the repeated application of high-volume stimulus during resistance training sessions maximizes the anabolic responses (higher protein synthesis) due to the greater metabolic stress generated (Schoenfeld, 2013). Finally, these authors also refer to the existence of a hypertrophic adaptations plateau, advising that training above this level could generate overtraining. Although resistance training volume seems to present a positive dose-response relationship, further research is needed to clarify the level over which there is a plateau, which is currently not well understood. These meta-analyses included in this umbrella review showed some limitations—authors highlight the scarce number of studies that included 4–6 sets per exercises as training variable, as well as the use of indirect measurement methods (e.g., BodPod) to assess the muscle gains in some of the includes studies.

### Frequency

Closely related to volume, frequency appears to be a key variable for hypertrophy gain, which refers to the number of resistance training sessions performed or the number of times a specific muscle group is trained in a given period of time, usually a week (Schoenfeld et al., 2016c). To determine the effects of resistance training frequency on hypertrophic outcomes, Schoenfeld et al. (2019a) observed that when volume is equated, frequency does not significantly impact muscle hypertrophy. Instead, the authors reported that a significant effect favoring higher frequencies was observed when volume was not equated. This could be due to the fact that by maintaining the volume, increasing the weekly frequency allows maintaining the intensity of the effort optimizing recovery between sessions. However, it has been observed that using high training frequencies combined with high intensities can lead to a rapid decline in performance and an increased risk of overtraining (Fry et al., 1994). Therefore, periodizing frequency and/or including periods of low frequency on a regular basis (i.e., tapering periods) can help to maximize hypertrophy responses and to reduce the potential for overtraining, but more research is needed to verify this hypothesis (Fry et al., 1994). Despite these results, training frequency can be a useful strategy to increase the overall training volume, a variable that has shown a dose-response relationship with hypertrophy (Schoenfeld et al., 2017a). In addition, variations in inter-individual responses to training frequency has been observed, so individualization of the training program is essential to maximize the hypertrophy potential of each participant (Haff and Nimphius, 2012).

The main limitations observed in the review in our hands, which examined the effect of training frequency on hypertrophy, were that the direct measurements were only carried out on the thighs and arms, so the results cannot be extrapolated to other muscle groups; it was not possible to compare the effect of training frequency between multi-joint and single-joint exercises as well as the effect of participants' age on chronical adaptations.

### Intensity

This variable is considered one of those with the greatest effect on hypertrophy responses (Fry, 2004). In this regard, each percentage of the 1RM is related to a certain number of maximum repetitions to be performed (Brzycki, 1993), traditionally categorized into low (<30% 1RM, >20 reps), moderate (30–70% 1RM, 11–20 reps) and high (>70% 1RM, <11 reps) ranges (Soriano et al., 2015, 2017). Consequently, each percentage of 1RM is associated with a different energy system and fatigue level (Sánchez-Medina and González-Badillo, 2011), impacting the extent of the hypertrophic response (Schoenfeld, 2010). Traditionally, training using high loads with a moderate number of repetitions (i.e., 80% 1RM, 8–10 reps) has been considered as a key strategy to optimize the muscle gains (Kerksick et al., 2009), based on the existence of an intensity threshold from which the increase in metabolic stress improves the hypertrophy response, allowing the recruitment of high-threshold motor units, which is not possible with the high repetition range (Schoenfeld, 2010). However, there is a lack of evidence to objectively establish the balance between external load, metabolic stress and hypertrophy responses. Schoenfeld et al. (2016a) performed a meta-analysis aiming to compare the effects of low- vs. high-load training in enhancing post-exercise muscular hypertrophy, who observed that resistance training programs using loads < 60% 1RM allows to achieve hypertrophy levels similar to those achieved with high loads (≥65% 1 RM) in untrained individuals, although they observed a trend toward greater hypertrophy using high loads (difference = 0.43 ± 0.24; CI: −0.05, 0.92; *p* = 0.076), (Schoenfeld et al., 2016a) which supports the established guidelines for hypertrophy training (loads > 65% 1RM) (Kraemer and Ratamess, 2004). These similar hypertrophy responses reported in this review could be due to several factors such as the training level of the participants involved in the studies, the length of the training process, the type of exercise utilized, rest interval, and training frequency. In a second meta-analysis, Schoenfeld et al. (2017c) observed similar hypertrophy changes after the use of high or low loads when muscle failure was reached. Conversely to the first reported meta-analysis, this study did not observe a superior trend toward the use of high loads. These authors suggest that training with low loads implies a higher level of discomfort, although this did not imply a lower adherence to it compared to that reported in programs with high loads. Finally, Grgic (2020) conducted a new meta-analysis on this topic and observed non-significant differences in hypertrophy when comparing the effects of low-loads vs. high-loads (performed to momentary muscular failure) in both type I and type II muscle fibers. Additionally, this author reported a 95% confidence and prediction intervals very wide, so there is a clear need for future research on this topic. These results suggest that the selection of the load to use within a strength training program whose objective is to increase muscle mass should be made based on individual criteria (e.g., training status of the participants, length of the training process).

A limitation of these meta-analyses (Schoenfeld et al., 2016a, 2017c) is the level of the participants (untrained individuals with minimal research on trained individuals) which difficult to extrapolate the results to trained athletes. In addition, few participants were involved in the included studies and the length of the protocols in some studies was a bit reduced.

### Contraction Type

Traditionally, it has been assumed that eccentric contractions promote greater gains in muscle mass compared to concentric contractions (Hortobágyi et al., 2000), based on the idea that the mechanical stress placed on the eccentrically contracted muscles triggers a progressive activation of genes responsible for cellular growth and development, which is not possible by concentric or isometric actions (Chen et al., 2002; Barash et al., 2004). Additionally, some authors have suggested that eccentric actions promote a more rapid protein synthetic response and greater increases in anabolic signaling (Franchi et al., 2014), generated by the result of the increase muscle damage (Schoenfeld, 2012). To get a more comprehensive knowledge about the superiority of eccentric contractions compared to concentric contractions in muscle gains, Roig et al. (2009) observed that eccentric exercise is more effective than concentric exercise in increasing muscle girth mainly due to the higher absolute loads imposed during eccentric contractions. However, these authors also indicated that concentric training performed separately can promote increases in muscle mass. Schoenfeld et al. (2017b) performed a meta-analysis and confirmed the advantage of eccentric contractions for increasing hypertrophy, although this advantage was relatively small (eccentric training 10% vs. concentric 6.8%). These differences could be explained because of the higher force and mechanic load generated during eccentric training compared to concentric when the same repetitions number is performed (Schoenfeld et al., 2017b). However, when mechanical work was equaled, the results obtained were inconclusive (Hawkins et al., 1999; Moore et al., 2012). Additionally, Schoenfeld et al. (2017b) also observed that concentric contractions induced hypertrophy gains in the middle portion of the muscle, while eccentric contractions have a greater effect on the distal portions, possibly due to localized muscle damage along the fiber produced by non-uniform muscle activation of eccentric contractions. Due to the different responses of both contractions, it seems appropriate to combine both types to optimize the hypertrophy response (e.g., using technologies that allow this) (Beato and dello Iacono, 2020). Finally, these authors found that eccentric training produced greater hypertrophy of type II fibers than concentric, which could be explained because eccentric contractions preferentially recruit high-threshold motor units, which contain more type II fibers (Beato and dello Iacono, 2020). However, the mechanism by which such a fiber type preference exists is not clear.

The comparison between adaptations on hypertrophy caused by concentric and eccentric contractions have been limited by the difficulty of isolating them from human movement, which implies a cyclical repetition of both types of contraction; the uncertainty to know the intensity implied by an external load during an eccentric contraction; and the specificity of resistance training regarding speed and mode of contraction (Roig et al., 2009). Therefore, it seems necessary to delve into the possible relationship between the effects on hypertrophy and the type of contraction, differentiating between body hemispheres, different muscle regions, the role of induced muscle damage in the increase in hypertrophy, the influence of the angle of pennation and the length of the fascicle and the comparison between types of contractions equating mechanic load (Roig et al., 2009; Schoenfeld et al., 2017b).

### Repetition Duration

Training with loads lower than 80–85% 1RM allows the trainee to voluntarily modify the tempo of the lift (Bamman et al., 2001), an action that reduces the velocity of the lift by increasing the mechanical tension manifested by the muscle (Westcott et al., 2001), thus promoting a greater hypertrophy response (Schoenfeld et al., 2015). In this regard, Schoenfeld et al. (2015) conducted a meta-analysis and observed similar gains in hypertrophy when training with repetition durations ranging from 0.5 to 8 s (to concentric muscular failure). However, it was also observed that training at volitionally very slow durations (10 s per repetition) is inferior from a hypertrophy standpoint. The authors speculate on the existence of a possible threshold velocity below which the hypertrophy response is impaired, since it could not be a suitable stimulus to recruit all motor units of a muscle—mainly high-threshold motor units (Keogh et al., 1999). Nevertheless, the training programs analyzed in this study were performed until concentric failure, which implies a progressive increase in fatigue along the set, reducing the motor unit recruitment thresholds, thereby enhancing muscle recruitment (Mitchell et al., 2012). From a practical perspective, a wide range of repetition durations can be used to stimulate hypertrophy, however, very slow repetitions (around 10 s) should be avoided. Considering that the evidence on this topic is limited, future studies on the effects of variation in the duration of repetitions must be performed in different contexts.

### Exercise Order

Multi-joint exercises are those that recruit one or more large muscle groups involving two or more main joints, while single-joint exercises are those that involve smaller muscle groups involving a single main joint (Haff and Nimphius, 2012). Specifically, multi-joint exercises generate a significant stabilization of the body, involving numerous muscles that could not be stimulated by single-joint movements (Schoenfeld, 2010). However, the biarticular muscles do not receive sufficient hypertrophy stimulation in multi-joint exercises since, during their execution, these muscles maintain a relatively constant length. Therefore, single-joint exercises are necessary to achieve a better length-tension relationship (greater mechanical tension following the length-tension principle) and a greater electromyography activity (a possible greater motor unit recruitment) (Schoenfeld et al., 2019b). In this sense, to know the best organization of these type of exercises within a resistance training session seems to be a key factor in order to optimize the muscle mass gains. Nunes et al. (2020) conducted a meta-analysis to analyze the effects of exercise order on muscular hypertrophy and the obtained results indicated similar hypertrophy responses regardless of exercise order, although authors claimed that this finding should be viewed with caution. In the included studies in which model B ultrasounds were used, measurements were taken in muscles that were not the object of the investigation, that is, hypertrophy measurements were performed on muscles that were agonists in the single-joint but synergists in the multi-joint exercises (e.g., biceps brachia in a bicep curl and in a vertical pull). In the studies that used indirect measurement methods, no significant differences were found either, but these methods have low sensitivity to identify subtle hypertrophy changes (Haun et al., 2019). Currently, we do not have enough evidence to state proper guidelines and, therefore, more research seems necessary, including analysis of different exercise orders with exercises on the same target muscle in which it also acts as the main agonist, and not only as a synergist, as well as using direct measurement methods on specific muscle regions (which have greater sensitivity).

### Time of Day

Human motor performance varies depending on the time of day (Drust et al., 2005). The time of day in which maximum performance is reached is called acrophase, which is around 6:00 p.m. attending resistance training (Guette et al., 2005). In this regard, Grgic et al. (2019) concluded that the hypertrophy adaptations were similar regardless of the time of day the training sessions were located. These findings could be partially explained by the similar levels of p70S6K phosphorylation observed after strength training performed in the morning or afternoon (Mayhew et al., 2011). These results suggest that the time of day for strength and hypertrophy training should be based on personal preference, although more research appears to be needed to really verify if differences exist between training in the morning vs. evening hours. Future studies should consider the assessment of CSA at the muscle fiber level and individual responses to resistance training at different times of the day based on chronotype (morning or evening) and habitual sleep cycles.

### Periodization

Triplett and Haff (2017) define periodization as “*the logical and systematic process of sequencing and integrating training interventions in order to reach peak performance at appropriate times*.” Two of the most used periodization models in resistance training are the linear periodization model and the non-linear or undulating model. To compare the effects of these periodization models, Grgic et al. (2017) conducted a meta-analysis and found that at the same training volume, no significant differences were observed, although it cannot be guaranteed that the same occurs with other forms of periodization. With these results, the importance of training volume in modulating the hypertrophy response was once again confirmed (Schoenfeld et al., 2017a). For this reason, linear periodization does not seem to be the most appropriate since it ends with the minimum volume and it is suggested that the inverse linear periodization model, in which the intensity is decreased and the volume increases, seems to be a better alternative since the maximum volume would be at the end of the macrocycle (Prestes and Lima, 2009). Even in trained subjects, who tend to present an attenuated response to training, significant improvements in hypertrophy have been observed without having applied any periodization model, simply with an adequate progressive overload. This leads to questioning the need to implement periodization models in resistance training programs (Morton et al., 2016). A possible consideration when establishing a periodization model can be the motivational factor in order to ensure adherence to training, being linear periodization more suitable for individuals who want to record weekly or monthly progress; while undulating periodization might be recommended for those who enjoy more the variety of training or because they have different training aims (Grgic et al., 2017).

### Blood Flow Restriction

To optimize hypertrophy, different strategies related to resistance training have been implemented, highlighting the blow flood restriction (Slysz et al., 2016). This method is based on the decrease blood flow to a muscle by application of an external constricting device, such as a blood pressure cuff or tourniquet, to provide mechanical compression of the underlying vasculature (Slysz et al., 2016). In this regard, in with the aim to increase the knowledge about this technique, Slysz et al. (2016) assessed the effectiveness of blow flood restriction exercise on muscle hypertrophy compared to traditional resistance training. These authors observed that the addition of blow flood restriction to dynamic exercise training is effective for augmenting changes in muscle size, mainly when training programs last at least 8 weeks and cuff pressures > 150 mmHg are used. Accordingly, Lixandrão et al. (2018) demonstrate similar muscle gains for high-load as compared with low-load resistance training associated with blow flood restriction techniques. Even though occlusion pressure, which is highly dependent on the width of the cuff, has been considered an important variable in blow flood restriction due to its ability to modulate muscle adaptations, the results obtained show total independent of the absolute occlusion pressure and the width of the cuff used (Lixandrão et al., 2018). However, authors stated that the occlusion pressure has been shown to have a direct relationship with the perception of effort and suggest that blow flood restriction with low pressures is perceived as more comfortable and less physically demanding, being especially useful in individuals with low tolerance to physical stress. Future research on a possible preference of the blow flood restriction stimulus over type I fibers seems to be interesting.

### Methodological Quality of the Included Meta-Analysis

As assessed using the AMSTAR 2 checklist, the included meta-analyses are classified as moderate or high methodological quality. Despite the acceptable overall quality of the included meta-analyses, we noted, regarding to the GRADE quality assessment, that 6 out 14 meta-analyses did not reported information about the quality of the primary studies analyzed. This methodological issue shows the necessity of establishing clear and specific methodological guidelines to apply in resistance training meta-analyses to increase the robustness of the findings.

## Conclusions

Based on the available meta-analyses, it has been observed that volume, frequency, intensity, contraction type, repetition duration and the application of the restriction of blood flow conditioning hypertrophy adaptations in healthy subjects, being volume the only resistance training variable for which a dose-response relationship with hypertrophy adaptations has been observed. Conversely, other variables as, exercise order, time of the day and type of periodization appear not to directly influence the magnitude of muscle mass gains. These findings provide valuable information for the design and configuration of the resistance training program with the aim of optimizing muscle hypertrophy.

## Practical Applications

From the existing literature some recommendations must be considered when resistance training program focused on muscle mass gains are prescribed:

a) *Volume*: research has reported a graded dose-response relationship between resistance training weekly volume and muscle growth. Therefore, it would be recommended to prescribe 2–3 sets per exercise, covering at least 10 weekly sets for each muscle group, while greater weekly volume does not seem to offer additional hypertrophy benefits.
b) *Frequency*: although the modification of this variable does not directly influence hypertrophy gains, significant effect favoring higher frequencies was observed when volume was not equated, therefore training frequency can be used as a tool to modify the overall weekly training volume.
c) *Intensity*: the choice of light or heavy loads can be made depending on the characteristics of the subject, although always reaching or close to failure. It is appropriate to prescribe variations in the magnitude of the load (<60% 1RM and >60% 1RM), however higher load seem to offer greater adaptations.
d) *Contraction type*: it seems appropriate to combine both concentric and eccentric contractions to optimize hypertrophy response, however, it seems that eccentric contractions may offer some additional advantages compared to concentric.
e) *Repetition duration*: a wide range of repetition duration seems to be appropriated to stimulate hypertrophic adaptations such as 0.5–8 s, instead, longer duration (very slow movement speed) is counterproductive, therefore it should avoid extending the repetition duration beyond 10 s.
f) *Exercises order*: for the modulation of this variable, personal preferences or specific objectives must be addressed, such as deliberately overloading a specific muscle group. Currently, we do not have enough evidence to state proper guidelines and, therefore, more research seems necessary.
g) *Time of day*: for the modulation of this variable, personal preferences must be addressed since no evidence in favor of a specific time of day (morning vs. evening hours) have been found on hypertrophy adaptations.
h) *Periodization*: individual preferences must be considered when choosing the periodization model to use, but always respecting the training volume and progressive overload.
*Blood flow restriction*: it seems appropriate to use this technique in widely experienced subjects or in those who cannot use heavy loads (i.e., injured athletes).

## Author Contributions

RB-V and JR-G conceived the research idea, collected the included studies, and prepared the manuscript. All authors critically revised the manuscript, read, and approved the final version.

## Conflict of Interest

The authors declare that the research was conducted in the absence of any commercial or financial relationships that could be construed as a potential conflict of interest.

## Publisher's Note

All claims expressed in this article are solely those of the authors and do not necessarily represent those of their affiliated organizations, or those of the publisher, the editors and the reviewers. Any product that may be evaluated in this article, or claim that may be made by its manufacturer, is not guaranteed or endorsed by the publisher.
